# Limitations of short range Mexican hat connection for driving target selection in a 2D neural field: activity suppression and deviation from input stimuli

**DOI:** 10.3389/fncom.2015.00128

**Published:** 2015-10-20

**Authors:** Geoffrey Mégardon, Christophe Tandonnet, Petroc Sumner, Alain Guillaume

**Affiliations:** ^1^School of Psychology, Cardiff UniversityCardiff, UK; ^2^Laboratoire de Neurobiologie de la Cognition, UMR 6155, Centre National de la Recherche Scientifique, Aix-Marseille UniversitéMarseille, France; ^3^Faculté de Psychologie et des Sciences de l'Education, Université de GenèveGenève, Switzerland; ^4^Laboratoire de Psychologie Cognitive, UMR 7290, Centre National de la Recherche Scientifique, Aix-Marseille UniversitéMarseille, France; ^5^Department of Psychology, New York UniversityNew York, NY, USA

**Keywords:** Dynamic Neural Field, action selection, center-surround, spiking neuron model, global effect, deviation away, saliency map

## Abstract

Dynamic Neural Field models (DNF) often use a kernel of connection with short range excitation and long range inhibition. This organization has been suggested as a model for brain structures or for artificial systems involved in winner-take-all processes such as saliency localization, perceptual decision or target/action selection. A good example of such a DNF is the superior colliculus (SC), a key structure for eye movements. Recent results suggest that the superficial layers of the SC (SCs) exhibit relatively short range inhibition with a longer time constant than excitation. The aim of the present study was to further examine the properties of a DNF with such an inhibition pattern in the context of target selection. First we tested the effects of stimulus size and shape on when and where self-maintained clusters of firing neurons appeared, using three variants of the model. In each model variant, small stimuli led to rapid formation of a spiking cluster, a range of medium sizes led to the suppression of any activity on the network and hence to no target selection, while larger sizes led to delayed selection of multiple loci. Second, we tested the model with two stimuli separated by a varying distance. Again single, none, or multiple spiking clusters could occur, depending on distance and relative stimulus strength. For short distances, activity attracted toward the strongest stimulus, reminiscent of well-known behavioral data for saccadic eye movements, while for larger distances repulsion away from the second stimulus occurred. All these properties predicted by the model suggest that the SCs, or any other neural structure thought to implement a short range MH, is an imperfect winner-take-all system. Although, those properties call for systematic testing, the discussion gathers neurophysiological and behavioral data suggesting that such properties are indeed present in target selection for saccadic eye movements.

## Introduction

The ability to select important stimuli for further processing and action planning is a key function of brains of visually dominant animals. For instance, in primate visuo-motor systems only a small part of the retinal input benefits from a high spatial resolution; hence to select where to look is vital to extract relevant information from the environment. Points of interest have to be extracted from the overall visual input and, from those extracted points, only one can be selected at a time to orient gaze or attentional focus. Since Koch and Ullman (1985) it is thought that potential points of interest are evaluated through early visual processing and converge on a saliency map.

It has been suggested for a long time that a connectivity pattern of short range excitation and long range inhibition in topographically organized visual structures could achieve *saliency localization*–see blob detection models for computer vision (Bretzner and Lindeberg, 1998; Lowe, 1999; Kong et al., 2013) but also models of V1/LGN (Kang et al., 2003; Schwabe et al., 2006; Spratling, 2010; Zeng et al., 2011)–and *target selection* (Arai et al., 1994; Kopecz, 1995; Kopecz and Schöner, 1995; Trappenberg et al., 2001). This connectivity pattern is often referred as a Mexican hat (MH) or center-surround inhibition, and was already implemented in early Dynamic Neural Field (DNF) models (e.g., Amari, 1977). Recently, the relevance of such organization has also been underlined for action selection in artificial cognition (Erlhagen and Bicho, 2006; Richter et al., 2012; Sandamirskaya, 2014); hardware implementations have emerged (Millner et al., 2010) and are suggested to be an important milestone for developing complex cognition (Indiveri et al., 2009).

Among neural structures often modeled using a DNF with MH connectivity (which we will refer to as DNF-MH), a prominent example is the superior colliculus (SC), a layered structure at the roof of the brainstem implicated in the control of gaze and attention orientation (Robinson, 1972; Sparks, 1986, 2002; Guillaume and Pélisson, 2001; Munoz, 2002; Krauzlis et al., 2004). The superficial layers of the SC (SCs) receive afferents directly from the retina and also from visual cortex and show strong visual activations. The intermediate-deep layers (SCi) display premotor activity for gaze orienting and receive multisensory input from a range of sources including connections from the SCs as well as “top down” input from frontal cortex and basal ganglia. Both layers are topographically organized (retinotopic organization) and in register to one another. This neural structure is hence seen as a sensory-motor interface able to associate a motor command to visual information through connections between superficial and intermediate-deep layers (Isa, 2002), as well as through other input. While both layers have been assumed to have MH connectivity, most modeling has focused on the SCi (and hence on target/action selection rather than saliency). Results of SCi studies (electrophysiology: McIlwain, 1982; Munoz and Istvan, 1998; and anatomy: Behan and Kime, 1996; Meredith and Ramoa, 1998) were in favor of MH connectivity and also suggested that inhibition from a given site can concern very distant areas of the map. Hence, without more precise measures, it was assumed that the inhibitory influence was very large. Numerous models implementing long range inhibition (Arai et al., 1993; Kopecz, 1995; Kopecz and Schöner, 1995; Trappenberg et al., 2001; Wilimzig et al., 2006; Meeter et al., 2010; Bompas and Sumner, 2011; Marino et al., 2012) showed that it was successful for winner-take-all selection of a saccade target among several options.

However, the idea of long range inhibition in the SC has been challenged (Lee and Hall, 2006; Isa and Hall, 2009). Very recently, a clearer picture has been obtained. Phongphanphanee et al. (2014) using multi-electrode arrays on slice preparations of rodent SC evaluated the local connectivity in SCs and SCi. This study found MH connectivity only in SCs and that, in this case, the range of inhibition is relatively short (see below for details). In SCi, the excitation zone was at least as large as the area of inhibitory influence. The second main difference between the SCs and the SCi revealed by the study concerned the time course of their excitatory and inhibitory responses to a sustained stimulation: where the SCi was behaving as an accumulator, the SCs showed transient responses. Globally these results led the authors to conclude that the winner-take-all phenomenon is observed in the SCs and that it enables saliency detection. Phongphanphanee et al. (2014) wrote “The sensory layer (SCs) is optimized to localize the single most salient stimulus” (p. 2342). The SCi, in turn, would cascade activity from the SCs and integrate it with its other inputs to perform target selection. Importantly, the saliency selected and localized by the SCs can be translated into the winner of the SCi target selection when other target candidates are negligible. As stated above, numerous models of the SC were implementing long range inhibition to perform selection. The results of Phongphanphanee et al. (2014) call for an exploration of properties of map integrating MH with short range inhibition and temporal dynamics based on the SCs.

The aims of the present study were: (1) to test the capacity of such a DNF-MH with short range inhibition to perform reliable target selection and (2) to highlight its noticeable properties and its potential limitations in such a context. Importantly, those properties could represent testable predictions to address if the SCs—or any brain structure—performances are indeed driven by a short range MH. We implemented this type of DNF-MH in two dimensions with spiking neurons. We fed it with various types of input stimulation to assess the emergence of localized and stable clusters of firing neurons (a “spiking cluster”) that would represent saliency and/or target selection. We first explore the effect of stimulus size on the performance of the model. Second, we tested the model while two stimuli were presented at the same time and we measured their interaction while varying their weights and the distance between them.

To anticipate some of the key results, varying the size of a single stimulation led to bimodal activation and to center-surround interactions that could result in the complete suppression of any activity on the network. When two stimulations were used, phenomena of attraction, complete suppression, and repulsion were observed for different distances. Applied to target selection, those properties may represent detrimental phenomena: prior loci of interest extracted from feature maps could suppress themselves, or produce clusters of activity that are not localized on the stimuli of interest. Interestingly, we can link these properties with previous neurophysiological and behavioral studies. These links are extensively explored in discussion.

As a final note, although our rational is largely based on results obtained in the SC, especially the superficial layers, our results describe a set of phenomena possible for any DNF-MH implementation and usage.

## Materials and methods

### Overview of the model

**Figure 1:**
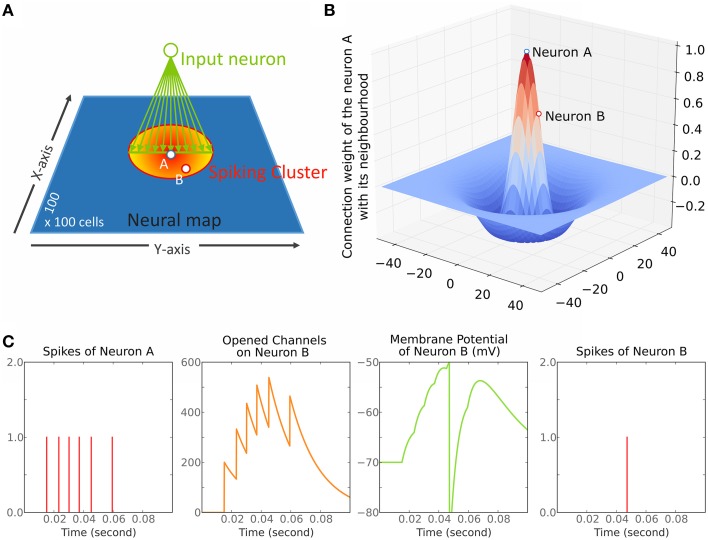
**Overview of the model. (A)** The model is a dynamic neural field (DNF) of 100 × 100 cells. The red to yellow circle represents the cluster of spiking neurons after stimulation of the neurons on the green line fed by the input neuron (green circle). This cluster forms a circle centered on neuron A. Neurons A (blue-white dot) and B (red-white dot) are marked in reference to **(B,C)**. **(B)** Illustration of the Mexican hat kernel. The graph shows the connection weight of neuron A with its neighborhood. The X- and Y-axis represent the distance from neuron A in number of cells; The Z-axis represents the weight of connection, a positive number is excitatory while a negative number is inhibitory (arbitrary unit). **(C)** illustration of Equation (1). Each spike of neuron A (panel 1, red bars) opens excitatory channels on the membrane of neuron B that close by themselves according to time constant τe (panel 2, orange curve). These opened excitatory channels raise the membrane potential of neuron B (panel 3, green curve). When a threshold (−50 mV here) is reached, a spike is triggered in the neuron B (panel 4, red bar).

The model is a simple network of neurons organized as one 2D layer of 100 × 100 cells (Figure 1A) and connected according to a 2D Mexican hat kernel (Figure 1B). Our model is close to those of Arai et al. (1993), Marino et al. (2012), Trappenberg et al. (2001) and Wilimzig et al. (2006). Nevertheless, the critical differences are that we implemented a MH with a short range of inhibition and that we used spiking neurons (Figure 1C) allowing to set up different synaptic decay times for inhibition and excitation (see Section Parameter Choice). Finally, we did not implement the logarithmic compression of space that is observed in the SC to remain general.

The model is implemented in Python 2.7 (http://www.python.org/) using the library BRIAN, a spiking neuron network simulator (Goodman and Brette, 2008, 2009). The code source for all the following simulations can be found at: https://github.com/Nodragem/SuppData-MHLimitations-Selection.

The spiking neuron model (Lapicque, 1907; Brunel and van Rossum, 2007) used here is a simplification of conductance-based integrate-and-fire (Hodgkin and Huxley, 1952; Shadlen and Newsome, 1998). Activity of each neuron of this network can be described with the following equations:

(1) $$\left\{ \begin{array}{l} \begin{array}{cc} \tau_{m}\frac{\partial V\left(n,t\right)}{\partial t}=-\left(V-V_{0}\right)-g_{e}\left(V-V_{e}\right)-g_{i}\left(V-V_{i}\right) & \left(\text{a}\right)\\ \frac{\partial g_{e}\left(n,t\right)}{\partial t}\text{​}=-\frac{g_{e}}{\tau_{e}}\text{​}+\alpha_{e}\sum_{n^{\prime }}\sum_{f}\delta \left(t_{n^{\prime }}^{f}-t\right)w_{e}\left(n^{\prime },n\right)+\alpha_{s}w_{s}F_{s} & \left(\text{b}\right)\\ \frac{\partial g_{i}\left(n,t\right)}{\partial t}=-\frac{g_{i}}{\tau_{i}}+\alpha_{i}\sum_{n^{\prime }}\sum_{f}\delta \left(t_{n^{\prime }}^{f}-t\right)w_{i}\left(n^{\prime },n\right) & \left(\text{c}\right) \end{array}\\ \text{ with}V=V_{r}\text{ if }V>V_{t} \end{array}\right.$$

Equation (a) describes the time course of the membrane potential *V* for all neuron n (Figure 1C, column 3). It goes toward its equilibrium *V*_0_ with a time constant τ_m_ when at rest while it goes toward *V*_*e*_ or *V*_*i*_ when *g*_*e*_ or *g*_*i*_ are different from zero. When *V* reaches a threshold *V*_*t*_ for a neuron *n*, a spike is emitted and *V* is reset to *V*_*r*_ for that neuron *n*. After it emits a spike, a neuron will be unaffected by any input during a refractory period of 1.5 ms. This refractory period limits the maximum firing rate to 600 Hz which is consistent with SC cell recordings for instance (Sparks et al., 1976; Anderson et al., 1998).

Equations (b) and (c) describe the time course of the opening of excitatory and inhibitory gates — *g*_*e*_ and *g*_*i*_ — on neuron n's membrane (Figure 1C, column 2). By default, *g*_*e*_ (respectively *g*_*i*_) goes to zero with a time constant of τ_*e*_ (respectively τ_*i*_) — the synapse decay time. For each time $t_{n^{\prime }}^{f}$ — corresponding to a spike *f* of a neuron *n*′ in the network — *g*_*e*_ (respectively *g*_*i*_) gets an immediate increase which corresponds to the weight connecting *n* to *n'* defined by *w*_*e*_ (respectively *w*_*i*_). Finally, one or more experimenter-controlled spiking neurons can be connected to the model through *g*_*e*_ (see Figure 1A). Their firing rate over time is controlled by a curve *Fs*; in that sense, they resemble electric stimulations used in neurophysiology and do not follow a Poison process. The connections to the network are defined within the unit interval with a matrix *w*_*s*_ and are modulated with α_*s*_.

The matrices of connections *w*_*e*_ and *w*_*i*_ are normalized between −1 up to 1: α_*e*_ and α_*i*_ are used to scale them to a relevant dimension for the network, its unit being millivolt. The matrices *w*_*i*_ and *w*_*e*_ are computed from a difference of Gaussians equation:

(2) $$\begin{array}{l} f(x,y|\theta_{x},\theta_{y},K,\beta )=(1+\beta )\text{exp}\left(-\frac{{\left(x-\mu_{x}\right)}^{2}}{2\sigma_{x}^{2}}-\frac{{\left(y-\mu_{y}\right)}^{2}}{2\sigma_{y}^{2}}\right)\\ \;-\beta \text{exp}\left(-\frac{{\left(x-\mu_{x}\right)}^{2}}{2K^{2}\sigma_{x}^{2}}-\frac{{\left(y-\mu_{y}\right)}^{2}}{2K^{2}\sigma_{y}^{2}}\right) \end{array}$$

**Figure 2:**
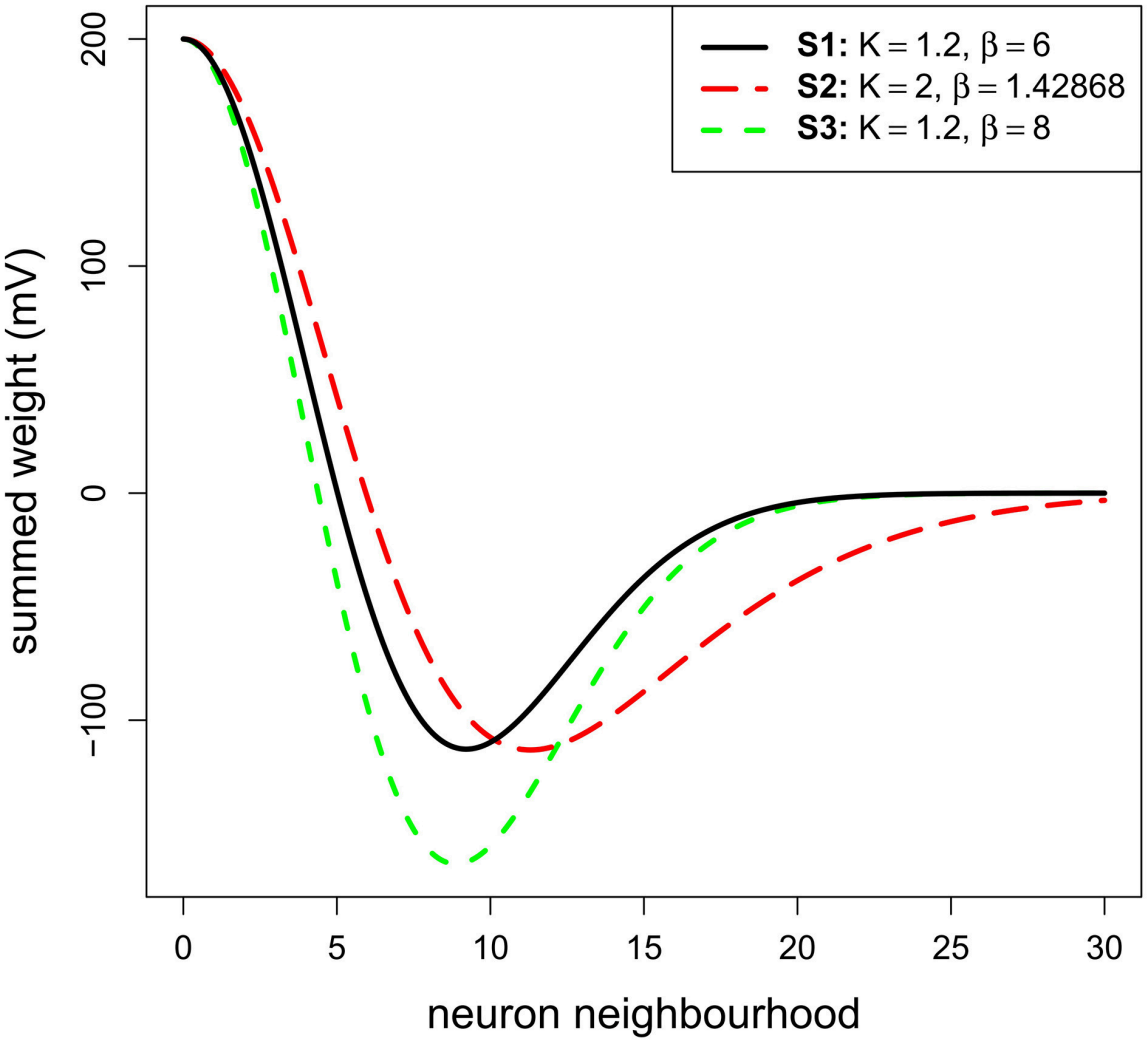
**The three Mexican hats tested in Simulation 1**. They correspond to the connection of a neuron n with its neighborhood (i.e., αe.we + αi.wi). They are only plotted on the X-axis and for one direction.

The resulting subtraction gives a Mexican hat curve (see Figures 1B, 2); the first term on the right hand side of Equation (2) is used as *w*_*e*_ and the second term as *w*_*i*_. The variables σ_*x*_/σ_*y*_ and *K*σ_*x*_/*K*σ_*y*_ define the standard deviation of the Gaussians. Thus, *K* is used to set up the *inhibition:excitation* extent ratio. β is a parameter controlling the depth of the inhibition.

### Parameter choice

All the parameters of the model are summarized in the Table 1. Parameters for which values are not given in the table have values varying in the different simulations and these values are to be found in the description of each specific simulation. The neuron parameters were taken from recent spiking neuron models of the SC (Lo et al., 2009; Morén et al., 2010, 2013) and adapted to obtain clusters that maintain a stable activity on the map for the range of stimulations we used. Concerning synaptic decay times, τ_*i*_ is superior to τ_*e*_ which is coherent with the observation of Phongphanphanee et al. (2014) during a sustained stimulation of the SC (see their Figure 7): indeed, **Figure 5E** of the present report shows that our model was able to reproduce a transient response of the membrane potential to a sustained input. However, to our knowledge the time constants τ_*i*_ and τ_*e*_ of actual SC neurons have never been specifically measured, which explains large differences in parameters values between the work of the two previous teams. By default, no noise will be introduced in the model. If a noise source is used, it will be stated in the text.

**Table 1:** **Model parameters and variables**.

**General parameters**	**Symbol**	**Value and unit**
Simulation time	None	200 ms
Map size	None	100 × 100 neurons
Simulation clock precision	None	0.01 ms
Recording clock precision	None	1 ms
**Neuron parameters**	**Symbol**	**Value and unit**
Membrane time constant	τ_*m*_	10 ms
Excitation time constant	τ_*e*_	3 ms
Inhibition time constant	τ_*i*_	10 ms
Potential threshold	*V_*t*_*	−50 mV
Reset potential	*V_*r*_*	−80 mV
Resting potential	*V_0_*	−70 mV
Nernst potential of excitation ions	*V_*e*_*	0 mV
Nernst potential of inhibition ions	*V_*i*_*	−80 mV
**Neuron variables**	**Symbol**	**Unit**
Membrane potential	*V*	mV
Number of opened excitatory channels	*g_*e*_*	No unit
Number of opened inhibitory channels	*g_*i*_*	No unit
**Mexican hat parameters**	**Symbol**	**Unit**
Depth of inhibition	β	No unit
Inhibition/excitation extent ratio	*K*	No unit
Standard deviation on Y-axis	σ_*y*_	Cells
Standard deviation on X-axis	σ_*x*_	Cells
Center position on X-axis	μ_*x*_	Cells
Center position on Y-axis	μ_*y*_	Cells
Matrix of positive connections	*w_*e*_*	No unit
Weight factor for positive connection	α_*e*_	200 mV
Matrix of negative connections	*w_*i*_*	No unit
Weight factor for negative connection	α_*i*_	200 mV
**External stimulus parameters**	**Symbol**	**Unit**
Spikes train	*F_*s*_*	No unit
Matrix of connections with the model	*w_*s*_*	No unit
Weight factor	α_*s*_	mV

Concerning the lateral connection parameters, we used values for *K* and β that were chosen based on previous physiological or modeling studies. *K*, corresponding to the ratio inhibition-extent/excitation-extent, was set to 1.2 to limit lateral inhibitory influence to a relatively small range consistent with recent results (see Isa and Hall, 2009 for a review). This ratio is similar to the value suggested by the SCs *in-vitro* study of Phongphanphanee et al. (2014). Indeed, they reported an EPSC half-width area of 130 μm^2^ and IPSC half-width area of 145 μm^2^ (see their Figure 4D and their text page 5; note also that Lee and Hall's (2006) *in vitro* study on rat SCi reported ratios of 500 μm/300 μm = 1.6 or 500 μm/400 μm = 1.25). The parameter β, corresponding to the strength of inhibition, was set at 6.0 in order to set the maximum inhibition weight at roughly the half of the excitation maximum weight to fit with the results of Arai et al. (1994) (see the black curve of our Figure 2—the minimum weight of the reference MH is at −100 mV for a maximum of 200 mV). Note that we test variations in these *K* and β values below.

Lastly, our parameters are chosen for the neural field to be bistable between the all-off state and a spiking cluster state. When a bump in the membrane potential reaches the threshold, the model generates systematically a stable and well-defined group of spiking neurons around the point which passed the threshold. We name this group a “spiking cluster” to distinguish it from bumps in the membrane potential. This spiking cluster is similar to a bump of activity in a population rate model, and being stable, it survives after we stop stimulating the neural field.

### Simulation set 1: Size variation of a single stimulus

In a first set of simulations we want to characterize the response of the selection map to stimuli of different sizes.

A range of stimulus lines of varying size (see below) was tested with the model. We ran three different sub-simulations (S1–S3): one testing the reference MH (see Materials and Methods) and two testing variations of it in order to make sure that the results are robust to moderate changes in the connectivity profile. The first sub-simulation (S1) implemented the reference MH (*K* = 1.2 and β = 6.0; see Model parameters). The second sub-simulation (S2) was conducted to test a larger extent of inhibition. *K* was fixed to 2.0, which covers the upper end suggested by data of Phongphanphanee et al. (2014). In order to only address the extent of inhibition, β was set to 1.43 with an optimization algorithm to keep the minimum of MH function (depth of the inhibition) similar to S1. The third sub-simulation (S3) was conducted with *K* = 1.2 and β = 8.0 to observe the effect of a stronger inhibition while keeping its extent constant. For the whole set of simulations in this part, σ_*x*_ and σ_*y*_ were fixed to 5 neurons, this was chosen to get relatively small MH lateral connections compared to the dimension of the model map. Given that the determinant factor is the relative size of the stimuli compared to the MH's size, having small connections allowed us to increase the range of tested stimulus sizes.

The map was stimulated with line-shaped stimuli of 20 sizes (2 neurons up to 42 neurons in steps of 2 neurons along the Y-axis). These line-shaped stimuli were defined by *I*_*s*_ = α_*s*_ × *w*_*s*_ × *F*_*s*_ as explained in the Equation (1b). The maximum size of 42 neurons represents less than 50% of the Y-axis size of the model map in order to limit border effects (map size = 100 × 100 neurons, see Table 1). The firing rate pattern over time of the external input, *F*_*s*_, was a Gaussian centered on 25 ms with a standard deviation of 80 ms and a maximum frequency of 400 Hz. The strength of the stimulus was α_*s*_ = 4000 mV. Finally, the duration of each simulation was 200 ms.

The results will be split in four parts. The first part focuses on the spatial pattern obtained on the map, the second part on the temporal dynamic. The third part investigates if our result would be different if using a sustained input (*F*_*s*_ = 400 Hz) instead of the Gaussian firing rate pattern aforementioned. Finally, the last part extends our results to bidimensional shape—replacing the line-shaped stimuli by squares and circles.

### Simulation set 2: Interaction of two stimuli

Our first set of simulations addresses the effect of stimulus size in a simple DNF-MH used as a target selection map. However, such a map is prone to receive many candidate points of interest from satellite structures feeding it. Our second set of simulations tests the behavior of our DNF-MH model when stimulated at two points with varying the distance and relative strength. In a comparison of our model with the SCs, this simulation is analogous to the *in-vitro* experiments conducted by Phongphanphanee et al. (2014) and by Vokoun et al. (2014) in which these two teams stimulated two points in the SCs varying the distance and the strength of stimulations injected in each point.

Two stimulation points, A and B, of size 2 × 2, are considered. Stimulation A is kept at a fixed location (*x* = 31; *y* = 51), while stimulation B is tested for distances from 2 to 40 cells with a step of 2. Stimulation A and B both have the same firing rate pattern as used in simulation 1. While the stimulation B is always connected with a weight of 4000 mV to the model, stimulation A is tested for 3 different weights: 1333 mV, 2000 mV and 4000 mV. We used the reference MH configuration (*K* = 1.2; β = 6.0) but in a larger implementation (σ_*x*_ = σ_*y*_ = 8.5 cells, compared to 5 cells in Simulation 1, to increase the MH size and hence virtually increase the granularity of our probing). The result we report here is the position of the spiking cluster nearest to stimulation B on the map. Its localization is defined by the center of gravity of its spike count over all the simulation. To control that border effects was not at the origin of the following observations, a control condition was run that tested the spiking cluster position for the different location of the stimulation B alone. The spiking cluster positions were well aligned with the stimulation B and suggest there is not border effect at those locations.

The results for the simulation set 2 will be split in two parts. The first part presents the results without including noise in the model. The second part tests if the results obtained for the condition 4000–4000 mV are robust to the addition of noise in the model and if they extend to a slight inequality in A and B intensity (3500–4000 mV). Precisely, the noise was added to the membrane potential and was following a normal distribution of standard deviation 4 mV.

## Results

### Simulation set 1: Spatial patterns

**Figure 3:**
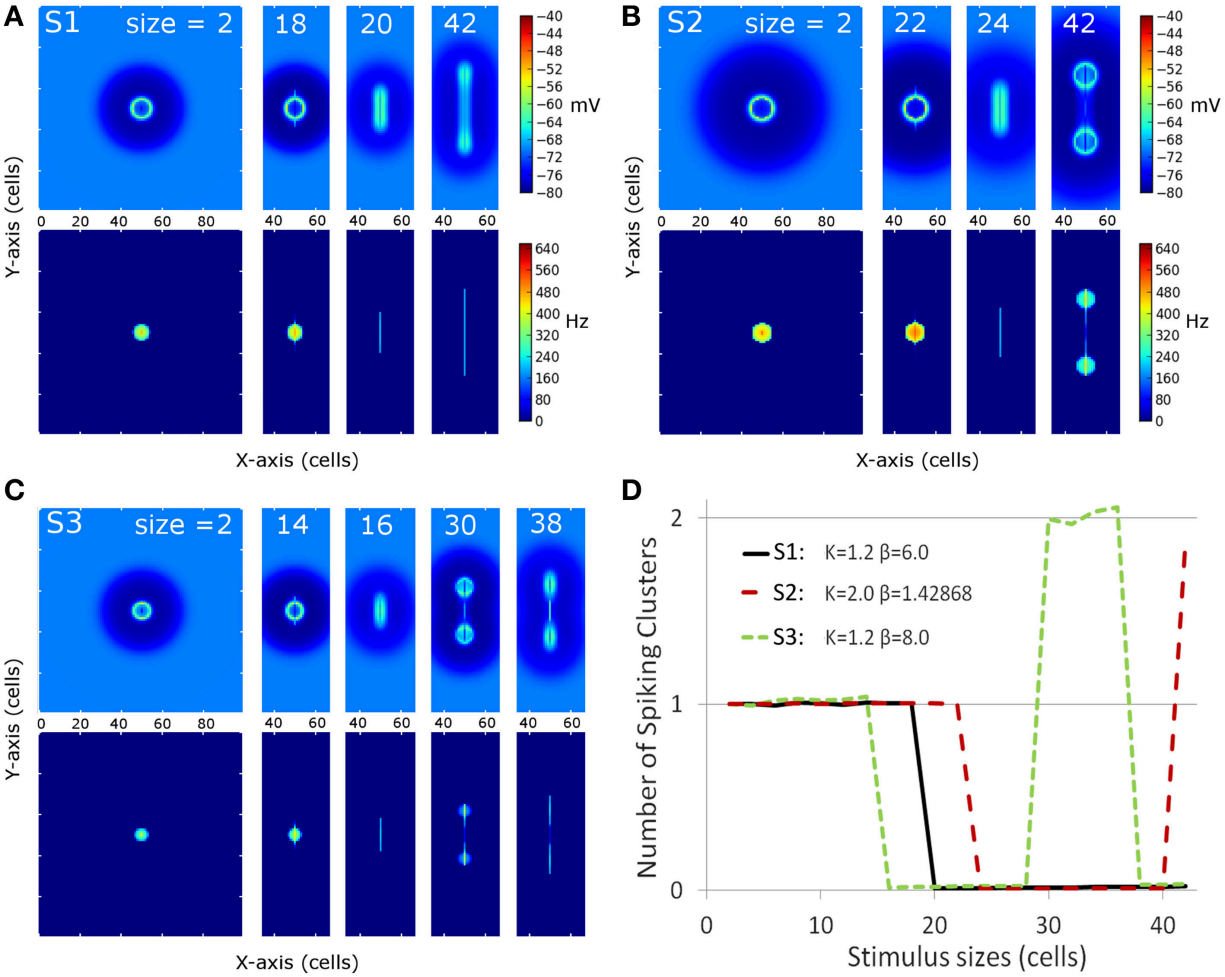
**Overview of the spiking clusters spatial distribution during S1, S2, and S3 (respectively A–C)**. The results are shown for the most informative stimulus sizes, which are different according to the set of simulations and are indicated on each column of the graphs. **(A–C)** On each picture, the top part shows average membrane potential during the simulation; the lower part shows average firing rate during the simulation. **(D)** summarizes the result: the number of spike clusters is computed as the sum of spikes on the map divided by the sum of spikes occurring for the first stimulus size (i.e. size = 2 is used as a reference).

Figure 3 shows membrane potential and firing rate for all neurons of the neural field for a subset of stimulus sizes for the 3 MH variants (S1, S2, S3, depicted in Figure 2). These values of membrane potential and firing rate were averaged over all the simulation time (200 ms). The represented line sizes illustrate the different observed activity patterns. Panel D shows the number of spiking clusters (see parameter section for definition) as a function of the stimulus size for the three MH variants, as further explained below.

For the first MH (S1, *K* = 1.2 and β = 6.0, Figure 3A and black line in Figure 3D), a unique circular spiking cluster located on the center of the stimulus line was observed from the smallest size up to the size of 18. Despite the transient nature of the stimulation, the spike cluster persists during the whole duration of the simulation. On the contrary, from size 20 to the largest tested size (size 42), no spiking clusters appeared on the firing rate map: a complete activity suppression was observed. It can be noticed that on the membrane potential map, the activity appears equally spread for size 20 while the activity is stronger on the extremities for size 42. This sub-threshold activity distribution suggests that the extremities could win the competition if the threshold was decreased.

In the second sub-simulation (S2, *K* = 2.0 and β = 1.43, Figure 3B and red line in Figure 3D), we observed similar results but the spiking cluster for small line sizes was larger and the complete activity suppression starts at a larger stimulus size. These two observations are to be related to the slightly larger excitation influence in S2 with respect to S1 (Figure 3). The main difference with S1 appears at size 42: the activity on the extremities was strong enough to give rise to two spiking clusters. Those two spiking clusters have a weaker average firing rate than the one observed for previous sizes; below we show this is due to a larger latency before the first spike rather than a lower firing rate once initiated (see next section).

In the third sub-simulation (S3, *K* = 1.2 and β = 8.0, Figure 3C and green line in Figure 3D), similar results as in S2 and S1 are found but with a smaller radius of the spiking cluster for small line sizes and a suppression that starts at a smaller stimulus size (size 16). Here, two spiking clusters were observed, as in S2, for stimulus size 30–36. However, S3 differs from S2 as a complete suppression was again observed for larger sizes than 36. When present, the two spiking clusters were on average weaker than the unique spiking cluster observed for smaller sizes, and again this is due to delay rather than firing rate once the spiking cluster occurs (see below).

Thus, complete activity suppression occurred for at least one range of sizes for each set of simulations. The stimulus size for which it appears is positively correlated with the size of the positive area of the MH used for these simulations.

Lastly, note that the stimuli tested were spatially homogenous: each point of the stimulus gave the same input to the map. This type of stimulation may favor complete suppression, and if noise were present in the network, it is conceivable that it could randomly favor the selection of a spiking cluster and hence eliminate the phenomenon of complete suppression. To test this hypothesis we added normally distributed noise in the membrane potential of all the units of the 2D network, using *K* = 1.2 and β = 6.0 (S1). The standard deviation of the noise was of 4 mV, which corresponds to a fifth of the distance between the resting potential and the threshold. The results were similar to those presented above. Hence, even with noise in the network, the phenomenon of complete suppression could be observed.

### Simulation set 1: Temporal dynamics

**Figure 4:**
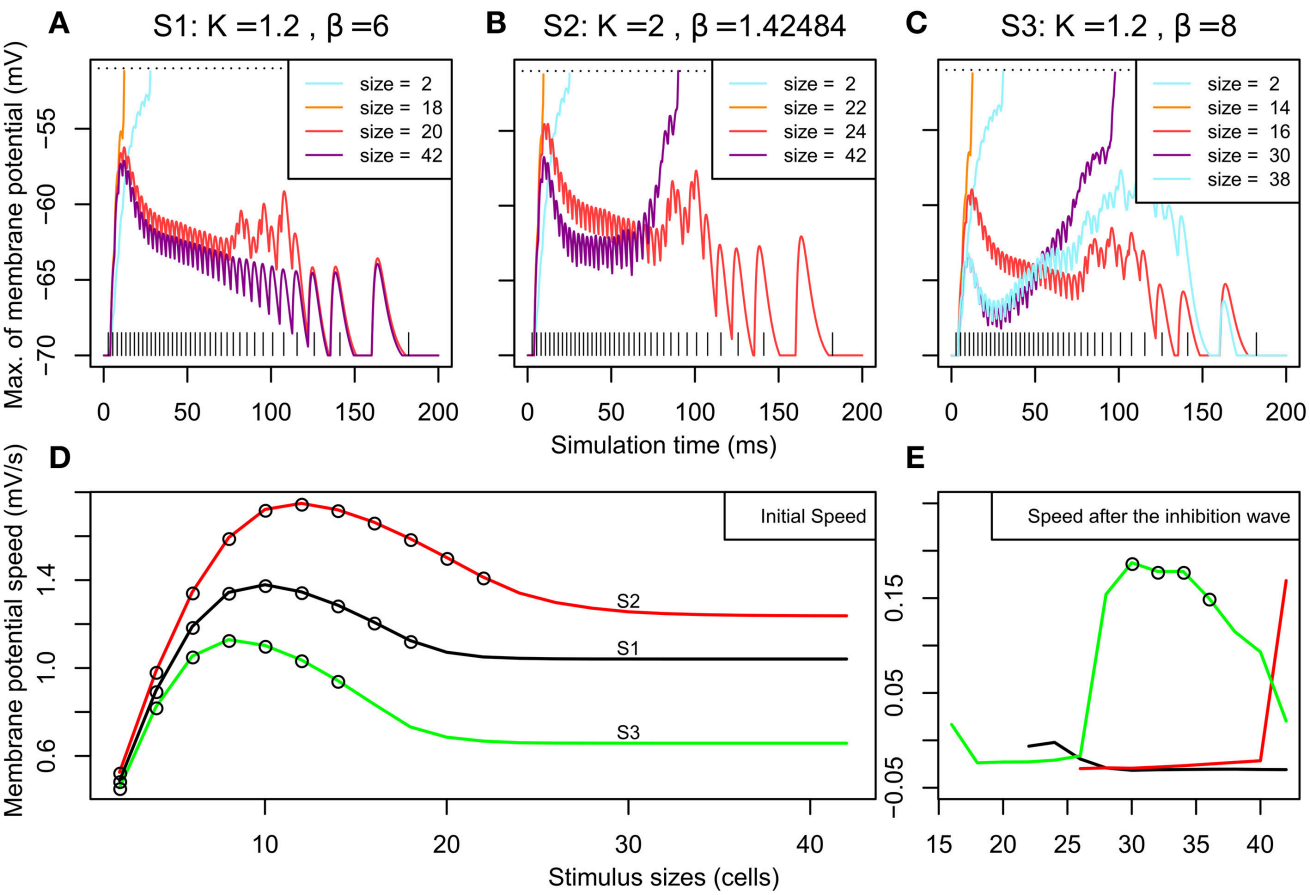
**Overview of the membrane potential dynamics during SA1–SA3**. **(A–C)** Effect of the stimulation on the neighborhood according to time. We report the membrane potential of the most excited neuron among the neurons situated along a line parallel to the stimuli and at 2 cells from it (*x* = 52). When a neuron on this line reaches its threshold (at −50 mV, see the dashed horizontal line), it means that a spiking cluster is created. **(A–C)** correspond to the 3 different MHs introduced in Figure 2; their parameters K and β (from Equation 2) are indicated. For each MH, we report the results for the sizes presented in Figure 3. The vertical lines at the bottom represent the input spike train. **(D)** Initial speed (averaged between 0 and 6 ms) of the membrane potential (in mV/s) according to stimulus size for SA1–SA3. The circles plotted on the curves denote that, for these stimulus sizes, the membrane potential reached the threshold before 30 ms and led to one spiking cluster on the neural field. **(E)** Speed of the membrane potential (in mV/s) between 30 and 90 ms – if the threshold was not reached during the first rise – plotted according to stimulus size. The circles plotted on the curves denote that, for these sizes, the membrane potential reached the threshold sometime after 30 ms and there are two spiking clusters on the map.

In simulations S2 and S3 larger stimuli could lead to two spiking clusters, which show a lower firing rate average than for the unique cluster appearing for smaller sizes. Figures 4A–C shows the evolution of the membrane potential for neurons just next to the stimulus line (see caption for more details) for the same sizes addressed in Figures 3A–C. It can be observed that the threshold to the first spike is reached much later for sizes giving rise to two spiking clusters (size 42 in SA2 and size 30 in SA3) when compared to sizes leading to one spiking cluster. In addition we have estimated the firing rate of the spiking clusters for the last 50 ms of each simulation: their firing rate does not change between stimulus size (550–600 Hz for S2, 350–400 Hz for S3,). Hence the change in firing rate average observed in S2 and S3 was the result of the change in latency for the membrane potential to reach the threshold.

**Figure 5:**
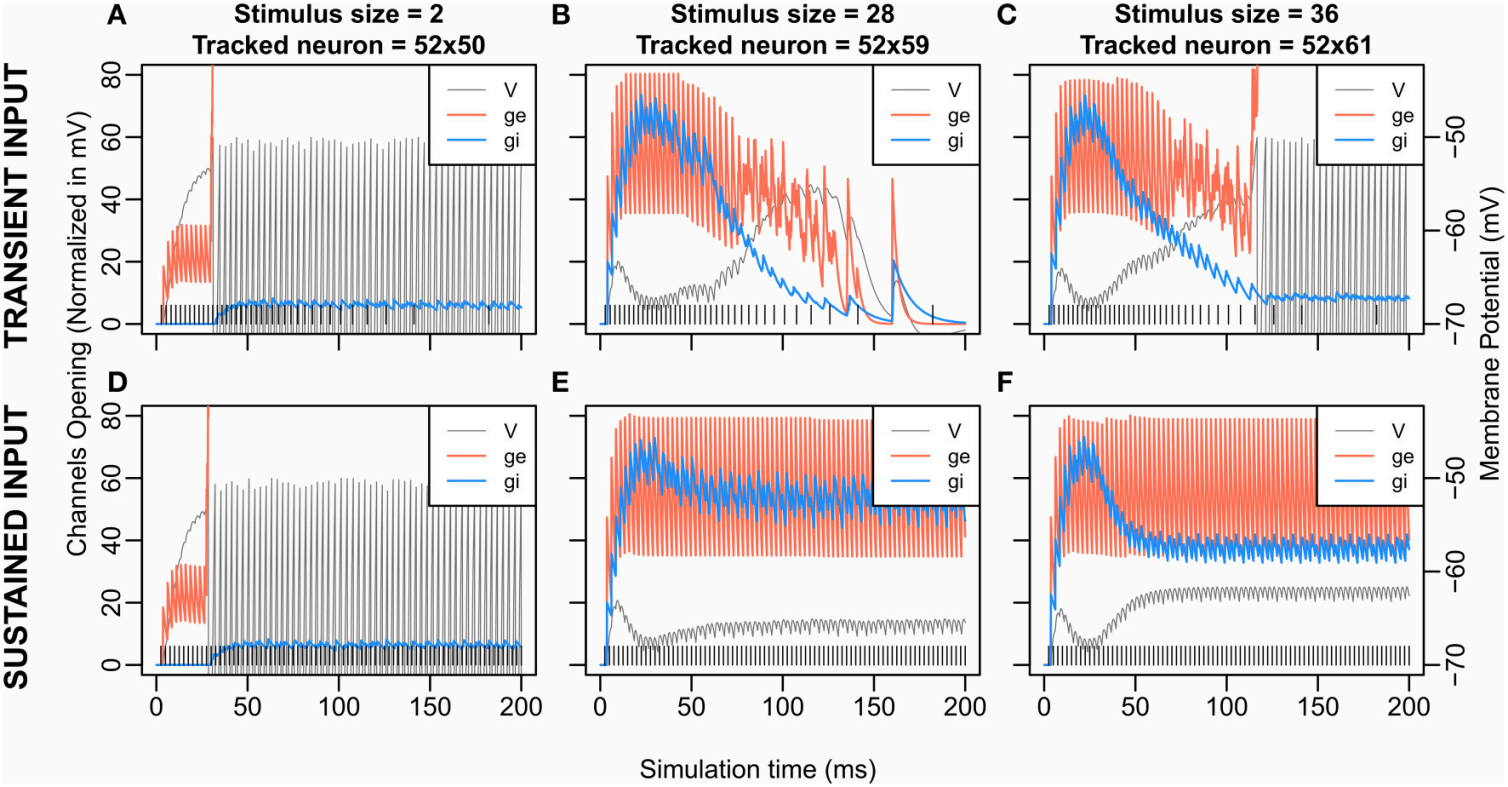
**Excitatory and Inhibitory Channels opening and membrane potential according to time for transient stimulation (upper graphs) and sustained stimulation (lower graphs)**. Data from S3 (*K* = 1.2, β = 8.0) are shown here; similar curves can be obtained from the other conditions. The transient input **(A–C)** is the one used in the previous simulations and corresponds to a Gaussian (see *F*_*s*_ in Inputs and Methodology). The sustained input **(D–F)** set *F*_*s*_ = 400 Hz, e.g., the firing rate of the input stimulation is at 400 Hz and constant over the time. The vertical lines at the bottom represent the input spike train. The tracked neurons all come from the row at 2 cells from the stimulated neurons (*x* = 52). At each timepoint, the following measures are extracted from the neuron that has the maximum membrane potential among the tracked neurons. The curve “V” is the evolution of the membrane potential over the time. “ge” or “gi” describe the evolution of, respectively, the number of excitatory or inhibitory opened channels on the neuron's membrane. However, for the sake of comparison, the number of opened channels ge and gi are multiplied by a scaling factor. Indeed, for any value of V: |*V*_*e*_-V| > |*V*_*i*_-V| where *V*_*i*_ and *V*_*e*_ are, respectively, the inhibition and the excitation equilibrium. Thus, an excitatory gate that opens always has more effect on V than an inhibitory gate. The scaling factors represent this difference by being $\left|V_{e}-\bar{V}\right|$ for ge and $\left|V_{i}-\bar{V}\right|$ for *g*_*i*_, with $\bar{V}=\left(V_{t}-V_{0}\right)∕2$.

We can observe in all the simulations (Figures 4A–C, all curves) an early rise of membrane potential. This early rise is at the origin of all single spiking clusters observed in Figure 3. Figure 4D shows the speed of this early rise (for the interval between 0 and 6 ms) for all stimulus sizes. This speed increases until an optimal size (10, 12, and 8 cells for S1, S2, and S3 respectively) and then decreases to a plateau. The obtained curve is analog to what is found with the firing rate of neurons in surround suppression literature (Sceniak et al., 1999; Schwabe et al., 2006). Empty circles on the curves indicate that the threshold is reached before 30 ms (i.e., a single spiking cluster is observed). Hence we can see that close to the stimulus size corresponding to the beginning of the plateau, the initial wave of excitation starts to fail to reach spiking threshold. Interestingly, in those conditions (size 20 and 42 of Figure 4A for instance), we can observe that the early rise is transient. This transient nature will be explained below with Figure 5 showing the dynamics of excitatory and inhibitory influences. Interestingly, this transient rise in the membrane potential echoes the transience observed by Phongphanphanee et al. (2014) in the SCs as previously mentioned (see their Figure 7A, left).

The two spiking clusters for larger stimuli occurred through a late second rise in membrane potential after 50 ms (e.g., size 42 and 30 for S2 and S3). By observing the curve for size 20 in S1 Figure 4A, we can see that a late rise in the membrane potential occurs also for intermediate sizes, but insufficiently to produce late spiking clusters (see also size 24 for S2 and sizes 16 and 38 for SA3), this corresponds to the complete activity suppression shown on Figure 3. To examine this further, Figure 4E plots the mean speed of the membrane potential averaged between 30 and 90 ms to illustrate how the second rise varies over stimulus size. The time window used catches the variation for S3 especially well showing that the second rise of the membrane potential, like the first, also has an optimal stimulus size after which the rise speed decreases again and it fails to reach threshold (compare with the Figure 3D).

### Simulation set 1: Effect of input dynamic

To get a better view of the dynamics of inhibition and excitation, we compared these dynamics for a sustained input at 400 Hz to those for the transient input with a Gaussian profile as previously used. Figure 5 shows the membrane potential of the neuron showing the largest hyperpolarization near the stimulus in these two cases (Figures 5A–C for transient input and Figures 5D–F for sustained input) obtained with parameters of S3 (*K* = 1.2, β = 8.0). It highlights that the dynamic of the initial transient rise in membrane potential comes from a delayed wave of inhibition (Figures 5B,C,E,F). Indeed, the weight of excitation is twice larger than the weight of inhibition, giving an initial advantage to the excitation which the inhibition later catches up due to its larger decay time constant. Note that this wave of inhibition comes from remote units (see Figure 2) and, so, does not appear for small size (Figures 5A,D).

With the sustained input, we still observe a second rise in the membrane potential; the inhibition curve still decreases after having overtaken the excitation. As our recording takes place toward the extremities of the stimulus—near the potential winner loci—this decrease of inhibition thus comes from the decrease of activity of the middle of the stimulus (see Figure 3B size 42, and Figure 4C size 30, 38) silently losing the competition at sub-threshold level.

For a stimulus size of 36, no spike cluster is observed in this sustained input condition in opposition with the transient stimulus condition (Figures 5C,F). As mentioned above, the second membrane potential rise is weaker when using the sustained stimulation. Counter-intuitively, this suggests that to decrease or to stop the stimulation input—with the transient stimulation—helped the membrane potential to reach the threshold. Two pieces of explanation are that (1) as the neurons are excitatory coupled, the most excited regions of the stimuli self-sustain their firing longer than the others when our input stops, (2) the most inhibited regions lose their only source of excitation when our input stops. Then to stop or decrease the input signal can accentuate disequilibrium in the competition and facilitate a target selection outcome.

### Simulation set 1: Generalization to 2D stimulus shapes

Our DNF-MH model of target selection map shows a phenomenon of total activity suppression related to stimulus size for 1D stimulus. Here, we generalize our observations to 2D stimuli by testing the behavior of the model when stimulated with a circle and a rectangle of varying size. We used the reference MH (S1, *K* = 1.2 and β = 6.0).

**Figure 6:**
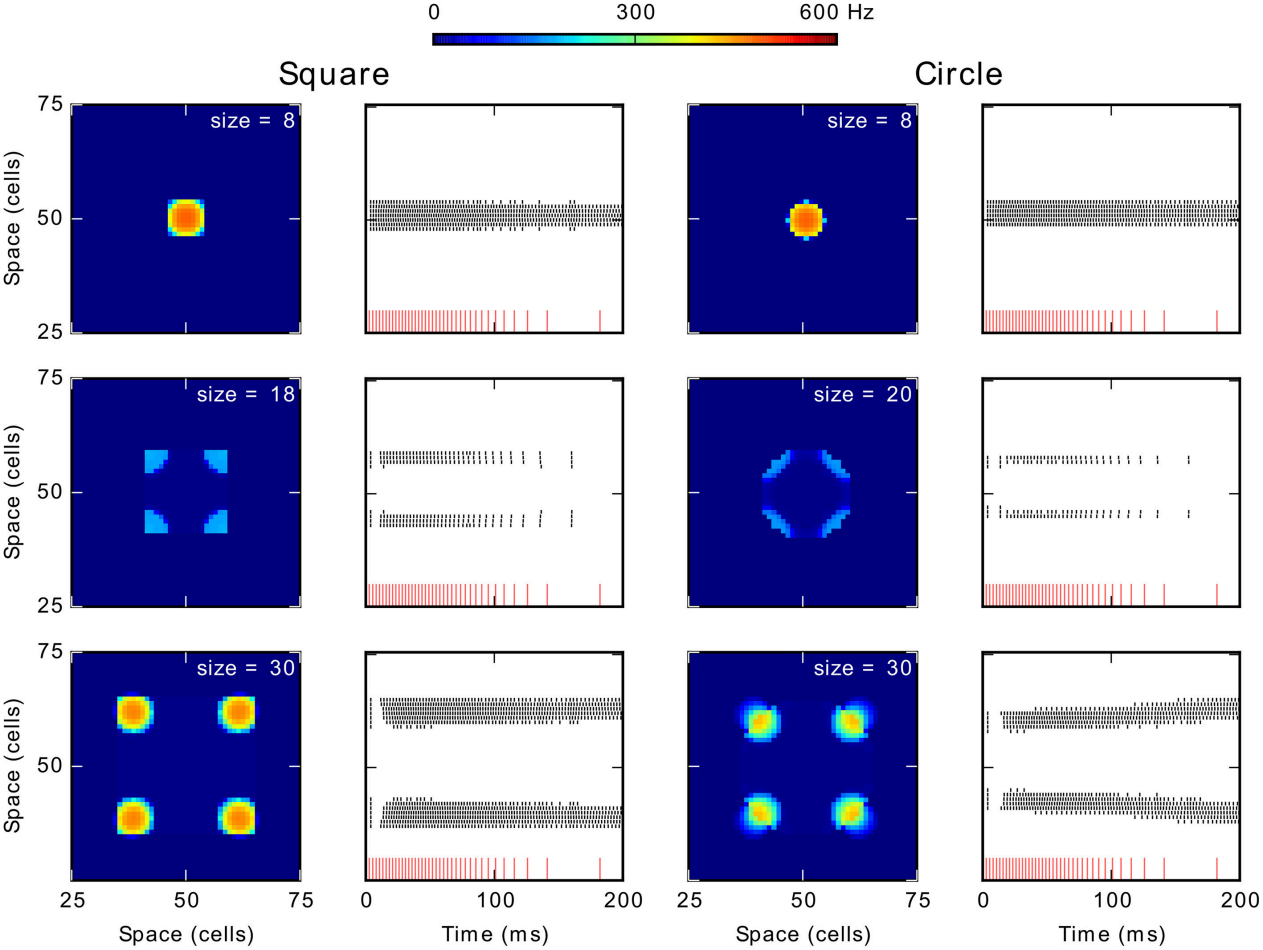
**Generalization of the effect of size to 2D stimulus shapes**. Column 1 and 3 show the average spike frequency (firing rate) in the neural map in Hz. The column 2 and 4 show the spikes train of neurons according to time. The red vertical lines at the bottom represent the input spike train. The recorded neurons are those forming the diagonal of the neural field from the position (25, 25) to the position (75, 75).

Figure 6 shows results obtained for these tests conducted with 2D shapes. Columns 1 and 3 show average firing rate over the simulation period and columns 2 and 4 contain the spikes train of neurons on the diagonal of the map. Similar phenomena of activity suppression to those for the 1D stimulus are observed: spiking clusters did not emerge for size 18 for the square and for sizes 20–26 for the circle. Additionally, for further increases of size, several clusters appear: from sizes 20 (square) and 28 (circle) four spiking clusters emerged (Note that activity for the circle segregates into 4 regions because the pixelation of our map, theoretically no point on a disk would be advantaged on a continuous competition field). Especially, in the case of the circle, these clusters tend to move as if they are repulsed from the center (lower panel of the column 4). This repulsion becomes weaker with size until a new spiking cluster emerges at the center in addition of the four on its corners (not shown).

The spike trains (columns 2 and 4) also allow observing a latency increase for clusters appearance when increasing the size. For smaller sizes, below 18 (square), and 20 (circle), the unique cluster appears with almost no delay with respect to the onset of the stimulation. Conversely, when spikes appear for larger sizes, whether they finally disappear (size of 18 for the square or of 20–26 for the circle) or are part of stable spiking clusters (larger sizes), there is a short latency period of approximately 10 ms before their appearance. This latency increase is similar to the one observed when a 1D stimulus resulted in two clusters (see above) but of lower value: the latency increase for the 1D stimulus was around 70 ms. Finally, the first burst of spikes and the following gap (just at the beginning of the stimulation; middle and lower panels in the columns 2 and 4) can be respectively related to the initial rise of membrane potential and to the wave of inhibition seen with 1D stimuli. These differences can be explained by the greater number of neurons interacting, which speeds and strengthens excitatory and inhibitory influence. Hence, apart from this difference in the latency, results for 2D shapes are similar to those obtained for the 1D stimulus (line).

### Simulation set 2: Spatial interactions

**Figure 7:**
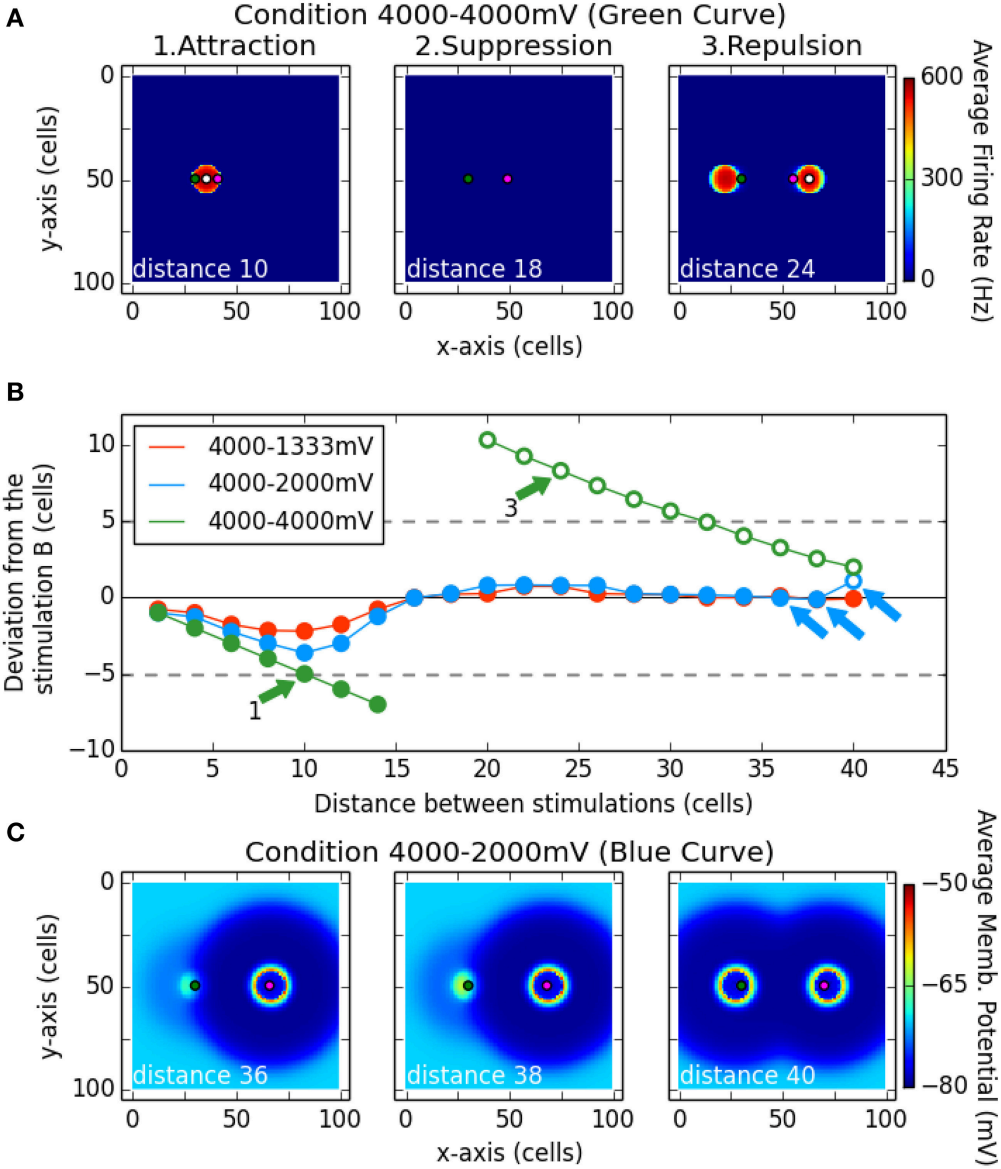
**Interaction between two stimulation induced bumps according to their distances**. The magenta dot in **(A,C)** represent the position of stimulation B, while the green dot represents the stimulation A. The white dot in **(A)** is the center of gravity of the spiking cluster the nearest from stimulation B. Subplot **(B)** describes the deviation of that center of gravity (white dot) from the stimulation B (magenta dot) on the x-axis. Filled dots denote there is only one spiking cluster on the map, while the unfilled dots denote there are two spiking clusters on the map. The simulation was run for different distance between the stimulation A and B (x-axis), and for different strength of the stimulation A (curves red, blue, and green). Note that subplot **(A)** shows an average of the firing rate over the simulation while subplot **(C)** shows an average of the membrane potential over the simulation.

Figure 7B shows a summary of the results for these simulations. The spiking clusters produced by the model indicate which locus has been selected as a target. Its deviation from stimulation B is shown as a function of distance between the two loci of stimulation. Negative values correspond to a deviation toward the locus of A. The three curves correspond to the three different intensity of stimulation tested for the point A (1333, 2000, 4000 mV; B is always stimulated with 4000 mV). Filled symbols indicate that only one spiking cluster was present on the network and open symbols that two clusters survived.

In the case of equal strength for both stimulations (green curve), for the first distances up to 14 cells, we observed one single resulting cluster (fusion phenomenon) that was in between the two stimulation loci (see panel A1 Attraction). That observation can be related to the activation merging found by Vokoun et al. (2014) in the SCs (see their Figure 3). Then for two following distances (16 and 18 cells), a complete suppression of activity on the map was observed (see panel A2 Suppression). Finally, from a distance of 20 cells up to the largest tested distance (40 cells), two clusters are produced and the closer to the site of B is repulsed in the opposite direction with respect to A (positive values on y axis of the panel B). The panel A3 allows us to see that the same repulsion was observed for the cluster close to the A site. This repulsion phenomenon decreased as the distance between the two stimulating sites increased.

When stimulation B was stronger than stimulation A (blue and red curves), in almost every case only one cluster was produced: a winner-take-all mechanism occurred and selected a locus near stimulation B. Nevertheless, a deviation toward stimulation A is still observed up to the distance 16 cells: the spiking cluster appears in between the two stimulations. Note here that the selected locus is closer to the strongest stimulation and that it gets closer when the latter gets stronger. That bias toward the strongest stimulus is also observed in Vokoun et al. (2014). For larger separation distances, the winning cluster remained localized near site B. This result goes in line with the results of Phongphanphanee et al. (2014): when the stimulations are close enough, an activation is present at A and B sites while when the stimulations are more distant, no activity is recorded close to the stimulation A (the weakest) and a normal cluster is observed close to the stimulation B (the strongest). Nevertheless, the winner-take-all mechanism is not perfect: the selected locus is near to B but not aligned with it. Indeed a deviation away from stimulation A occurred, similar to what we observed with equal strength simulations.

Panels C1 and C2 show this winner-take-all phenomenon. However, for the last tested distance with *A* = 2000 mV (40 cells), the activity at A escaped from the inhibition influence of the stimulation B and two clusters emerged (panel C3). This may be seen as a fail to select one target from the two input: the stimulation A overcomes the surround inhibition—which decreases with the distance in that range of distances—and stimulation B gives rise to its own spiking cluster. That does not occur for the condition 4000–1333 mV, where the stimulation A is too weak to overcome the inhibition even at such a distance.

### Simulation set 2: Tight competition and addition of noise

Our previous results for two stimulation inputs of exactly same strength show that for a given range of distances a complete suppression of activity is observed. This corresponds to a failure for the DNF-MH model to select only one target. One can suggest that this failure of the winner-take-all is due to (1) the absence of noise in our model or (2) the unnatural exact equality of the two stimulations in competition.

We tested here if the previous results, notably the suppression, can be obtained with the addition of noise and for close competition (3500 mV vs. 4000 mV).

**Figure 8:**
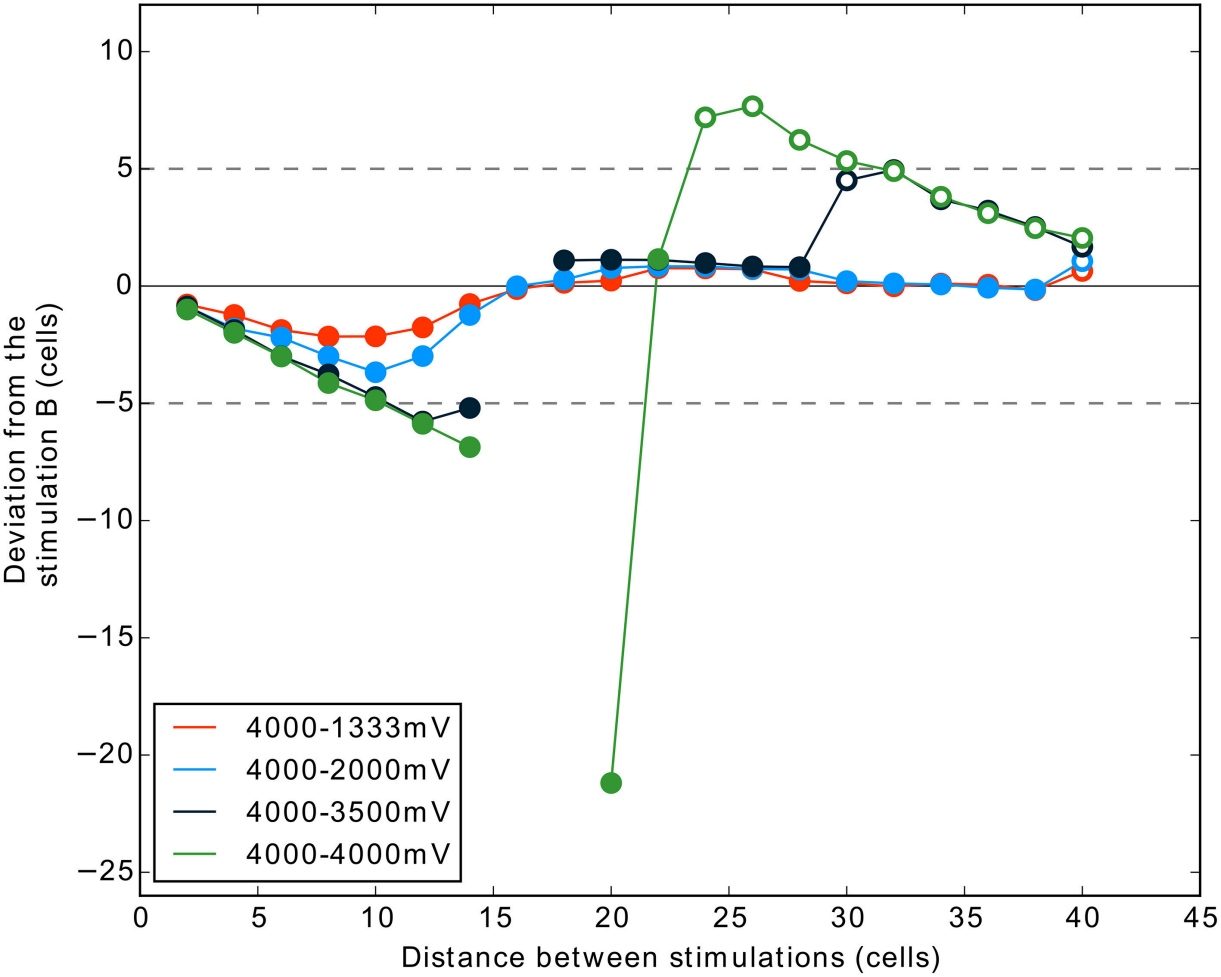
**Interaction between two stimulations according to their distances, with noise, and with an additional condition (4000–3500 mV) testing for tight competition**. Same description as for Figure 7B.

The Figure 8 shows the results of one simulation with these conditions. The results are strongly similar to those obtained in the simulation without noise. The addition of noise, even if it helps to get only one winner (compare distance 20 and 22 in Figures 7B, 8), does not prevent the occurrence of two-winners and no-winner situations in the 4000 mV–4000 mV condition. Interestingly, the condition 4000–3500 mV (dark blue curve) shows that we can also obtain activity suppression (Figure 8) when the two stimulations are not exactly of the same strength. This occurs for distance 16 and 18 cells, similarly to the equal strengths condition. However, in this case, the two-winners situation is not observed directly after the suppression phase. For some distances, the curve is similar to the one obtained in the condition 4000–2000 mV. Nevertheless, finally stimulation A succeeds to give rise to a spiking cluster because the inhibition from stimulation B gets smaller after a certain distance (refer to the shape of a MH curve, Figure 2). Here, stimulation A being stronger than in the 4000–2000 mV condition, it overcomes the inhibition of B (i.e., it results in two clusters) at a smaller distance.

## Discussion

The aim of the present study was to get further insight into the properties—and their consequences for saliency or target selection—of Dynamic Neural Fields based on a Mexican hat kernel (DNH-MHs) in the specific case of short inhibitory influence. Indeed, this type of lateral connection has been recently demonstrated for a classical biological example of DNF-MHs, the superior colliculus (SC; see Introduction). We designed a simple one layer model implementing the most recent data concerning lateral interaction in this neural structure (Phongphanphanee et al., 2014) and we tested its properties. We observed that certain stimulus sizes could lead through center-surround interactions to a complete suppression of the network activity, while larger sizes led to multi loci selection. This complete suppression, which led to no target selection, also occurred when two stimulations were presented simultaneously within a certain range of distances. For smaller distances, the model selected a position in-between, closer to the strongest stimulus (attraction/fusion), while for larger distances the model selected two loci that were deviated away from the stimuli positions (repulsion). We discuss these results of suppression and spatial deviation (i.e., attraction/fusion and repulsion) obtained here in view of neurophysiological, modeling and behavioral previous findings.

### Suppression phenomena: Neurophysiology results

It may seem counterintuitive to observe complete suppression on a saliency map for large stimuli of interest. This result can nevertheless be related to previous neurophysiological, modeling and behavioral findings in the visuo-oculomotor system and may help to disentangle unanswered questions.

Suppression phenomena in which larger stimuli produce lower activity than smaller ones are well described in sensory systems and especially in the visual system (Allman et al., 1985; Sèries et al., 2003). Most of the time a decrease in the response is observed (either a decrease in the frequency of the response or/and in the number of spike emitted; see Hubel and Wiesel, 1968), rather than a complete suppression as observed here. Nevertheless, phenomena of total suppression have also already been reported in physiological recordings. Goldberg and Wurtz (1972) showed a complete suppression of SCs response when increasing the size of a visual stimulus (see their Figure 4). Additionally, more recently, in a study on SCs receptive field, Wang et al. (2010) reported that the activity of SCs neurons was completely suppressed for large stimuli centered on the tested neurons (see their Figure 5). Our study brings some clues concerning mechanisms underlying these suppressive phenomena. Indeed, their neural substrates remain debated (Sachdev et al., 2012). The origin of the suppression is proposed to be due to (1) a decrease of feedforward activation (2) interactions involving local lateral connections or, finally, (3) feedback connections from higher areas. The present study confirms, on a theoretical ground, that center surround interactions in a single layer based on the most up to date physiological evidence from SC is sufficient to provide total suppression of the response for a certain range of stimulus sizes.

For any given surround suppression phenomenon, other observations in the present work provide predictions to test the hypothesis that it might be driven by short inhibitory lateral connections. First, increasing the size of a line stimulus should lead, after the suppression phase; to the reappearance of activation clusters on sites corresponding to extremities (see Figure 3). Second, this reappearance should be observed with a significant latency increase if a delayed wave of inhibition is present (see Figure 4). Third, when two stimulations are tested, maximal activity suppression should also be observed for a specific distance (see Figure 7).

### Suppression phenomena: Modeling results

Similar models with MH connections have already been suggested to reproduce surround suppression (Sceniak et al., 1999; Schwabe et al., 2006; Spratling, 2010). Only Sceniak et al. (1999) also showed total suppression (see their Figure 2F). Nevertheless, none of them were constructed with spiking neurons. Further, than the effect of the spatial organization of inhibition and excitation, our work gives an insight in how the *dynamic* of the inhibition and excitation can shape the suppression. In the present model, it is a delayed wave of inhibition—i.e., after an initial rise of membrane potential—which drives the surround suppression. A change of the inhibition time constant would modify the suppression effect. This dynamics of the membrane potential during the surround suppression phenomenon could be investigated in experimental intracellular recordings and, if matching those observed in the present study, be used to infer the inhibition time constant of the local circuitry.

Finally, our results suggest an optimal size of visual stimuli which minimizes the latency to trigger a spiking cluster (Figure 4D) in our target selection model. This is in line with the modeling work of Marino et al. (2012)—see their Figure 6F—who were working with a population rate model and an arbitrary threshold to trigger saccades. They also observed a U-shape relationship, but didn't observe a total suppression.

### Suppression phenomena: Behavioral results

If the suppression phenomenon observed in our model exists in the oculomotor system, this predicts that large stimuli will lead to fewer saccades with short latency than would small stimuli. Ploner et al. (2004) observed this type of effect in a behavioral study: saccades with short latency were less numerous for large targets (10°), whereas saccades with short latency were more frequent for small target sizes (1°). More precisely concerning this latency question, the U-shape curve for the relationship between the membrane potential evolution speed and the size of the stimulus (Figure 4D, see also Figure 6F of Marino et al., 2012) is in line with the relationship shown by Boch et al. (1984) between express saccades latency and the size of the target (see their Figure 5).

The observed suppression would similarly predict that larger distracting stimuli could paradoxically interfere less with saccades to a nearby target than might smaller distractors. Such a pattern was observed by Tandonnet et al. (2012). Their work focused on the Global Effect, which is the tendency for saccades to land in between to nearby visual stimuli (Findlay, 1982). Using a target-distractor couple, they found a U-shaped curve for such deviation while increasing the distractor size: first the distractor is too small to have a strong influence, then its increase in size makes its influence grows, but from a given size its influence begin to decrease. This loss of weight for larger stimuli could be explained by a decreased response in a saliency map such as the SCs or the LIP. Finally, the results of Van der Stigchel et al. (2012) consisting in a smaller extent of the global effect for large stimuli may also be explainable by a suppression of large stimuli. Note that while Tandonnet et al. (2012) observed the average shift of the landing positions, Van der Stigchel et al. (2012) observed the split from unimodal to bimodal distribution. All these results suggest that different degrees of suppression are observable at the behavioral level. It remains to be investigated whether total suppression phenomena can also be detected—for single stimulus, and for two stimuli.

### Spatial deviation: The fusion effect

Our DNF-MH demonstrates deviation of the spiking clusters from the initial input locations. Such deviation can be detrimental, for instance, when the DNF-MH is used as a target selection map which has to select among different points of interest sent by satellite structures. Indeed, with such deviation, the selected target would not correspond to any prior points of interest. We discuss here whether these deviations have already been observed at the neurophysiological, modeling or behavioral level.

When the model is stimulated at two nearby locations a single spiking cluster emerges in-between them. The cluster is closer to the stronger stimulation location—in proportion to its relative strength—and it is of the same width as spiking clusters induced by a single stimulation. This phenomenon of *attraction (and fusion)* was described for the first time by Amari (1977) in a DNF-MH based on a firing rate neuron model. In the context of the spatial working memory, Compte et al. (2000) proposed a model consisting in a one dimensional DNF with a MH connectivity pattern. Interestingly this group recently demonstrated that this model could lead to phenomena of attraction and fusion (Almeida et al., 2015). The findings of the present study extend these previous observations to a 2D spiking neuron networks.

On the behavioral side, the tendency for saccades to land in between two simultaneous and nearby visual stimuli is known as the Global Effect or saccade averaging (Findlay, 1982). Concerning the neurophysiological approach, Glimcher and Sparks (1993) showed that this fusion phenomenon could occur in the SCi when an intermediate saccade is made between two visual stimuli presented simultaneously. Edelman and Keller (1998) added that this could be the case for saccades of latency in the average range while two distinct bumps of activity would stand on the SCi for shortest latency saccades. However, whether a fusion of activity in the SCs or the SCi can explain the Global Effect is still matter of debate. Arai et al. (1994) implemented a saccadic system model using a DNF-MH to simulate the SC layers. Their model took into account the SC spatial compression and, in their test using fusion to explain the Global Effect, one can notice hypermetria (overshoot) of the output saccade (see their Figure 10). Katnani and Gandhi (2011) brought further insight for that result: when the DNF-MH phenomenon of fusion is applied in SC space (Note that the SCs and SCi are assumed to have to an equivalent mapping; cf. Schiller and Stryker, 1972), this would lead systematically to overshooting averaging saccades in external or retinotopic space. On the other hand, they demonstrated that a vector averaging of two steady bumps of the SC space would lead neither to a hypo- nor a hypermetria. They, however, note that if the phenomenon of attraction could lead to a wider bump of activity (wider on the axis formed by the two input stimulations, leading to an elliptic shape), the hypermetria would be corrected.

Recently, Vokoun et al. (2014) have reported in their work applying photodiode stimulations that on a coronal slice of the *superficial layers* of the rat SC- “simultaneous stimulation of two nearby sites resulted in a single, merged peak centered between the two sites.” They suggest that such a phenomenon could explain the Global Effect. Importantly, they observed that an activity bump induced by the simultaneous stimulation of two loci is wider than an activity bump induced by a single stimulation. That results interestingly echoes to a previous behavioral study observing that larger visual stimuli can lead to a wider distribution of saccade landing positions (Tandonnet and Vitu, 2013). Under the considerations of Katnani and Gandhi (2011) this spread of activation could correct the hypermetria issue discussed above, but it is important to note that a single layer bistable DNF-MH model such as ours could not replicate such a spread of activity–because the size of the spiking cluster is set by the width of the MH.

### Spatial deviation: The repulsion effect

When two clusters of activity were induced by two stimuli, they tended to deviate away from each other (see Figure 7). Here also both the early work of Amari (1977) and the recent study of Almeida et al. (2015) already observed this phenomenon in 1D models. Again our results allow to extend these findings to a 2D situation. To evoke this repulsion phenomenon, Amari (1977) explains that bumps of activity tend to climb up inhibition slopes. Then, the repulsion is reserved to MHs which have a range short enough to allow a stimulation to “climb” the outer inhibition slope of another.

Concerning the behavioral level, Wang et al. (2012) as well as Wang and Theeuwes (2014) suggest that if this phenomenon is present in the SC, it could explain the trends of saccade trajectories to deviate away from a distractor. Wang and Theeuwes (2014) also report a shift of the landing positions away from the previous fixation stimulus when varying its timing which might be explained by repulsion. However, to the best of our knowledge, repulsion in the bimodal distribution of landing positions to two simultaneously presented stimuli or in the internal representation of stimuli position has never been observed. This may be due to the difficulty to track back a phenomenon occurring in the SCs from behavioral data. For instance, the strongest repulsion effect we observed occurred when there are two spiking clusters emerging on the map. Nevertheless, if there is vector averaging downstream, at the behavioral level only a Global Effect might be observed.

Finally, at the neurophysiological level, Vokoun et al. (2014) studied activations in coronal slices of the superficial layers of the rat SC after concomitant stimulation of two sites. They did not observe any repulsion (nor any suppression) effect despite the exploration of numerous distances between the two stimulated sites. Hence, even though evidences have been found recently for a local Mexican hat kernel in the SCs (Phongphanphanee et al., 2014), the lack of concordance between the present study results and Vokoun et al. (2014)'s results questions if the SCs can be modeled with a simple DNF-MH (see also the end of the previous Section Spatial Deviation: the Fusion Effect). However, a possible alternative to explain this lack of concordance is that the coronal slicing used by Vokoun et al. (2014) may have damaged part of the lateral inhibition system altering the MH kernel, its size and its properties. Hence, further neurophysiological works are required to shed more lights on (1) the link between fusion of activity in the SC layers and Global Effect, and (2) on what extent those natural phenomena can be modeled with a simple DNF-MH.

## Conclusion

We constructed a DNF-MH integrating short range MH connections based on recent results obtained in the superficial layers of the SC, and we tested how it performs in very simple target selection tasks: (1) the localization of a single stimulus of different sizes; (2) the selection and localization of the strongest of a pair of stimulations.

Our work demonstrates that even a short range inhibition (i.e., only slightly larger than the excitation; ratio of 1.2) can enable a selection dynamic. However, it also highlights noticeable phenomena emerging from the model during those tasks: suppression, multi-spot selection, attraction/fusion, and repulsion. If the DNF-MH is used as a target selection map as it is thought to be the case for the SCs, such attraction and repulsion would impair the spatial precision of the selection while the suppression would delay or hinder selection. In short, those properties suggest that the SCs is an imperfect winner-take-all selection system. At the same time, those properties constitute a collection of testable predictions to verify this point and the pertinence of using a DNF with short range MH to model the SCs. In parallel, future modeling work may investigate whether the phenomena we observed survive more advanced implementations of the SC dynamics. Notably, (1) when one implements the transient visual burst dynamics in SCs; (2) when one implements the SCi layer and the motor executions. Finally, results obtained in the present study have been obtained with activity in the range of what can be observed in the SC (up to 600 Hz). Further work remains to be done to explore what would be observed in DNF with lower maximum frequency.

Interestingly, attraction and repulsion phenomena have recently been reported when using DNF-MHs in spatial working memory tasks, and they have been successfully related to actual behavioral imprecisions (Almeida et al., 2015). Those results support the point that DNF-MHs are imperfect winner-take-all systems and relevant models of biological networks at the same time.

### Conflict of interest statement

The authors declare that the research was conducted in the absence of any commercial or financial relationships that could be construed as a potential conflict of interest.
